# Modulation of Allergic Reactivity in Humans Is Dependent on *Schistosoma mansoni* Parasite Burden, Low Levels of IL-33 or TNF-α and High Levels of IL-10 in Serum

**DOI:** 10.3389/fimmu.2018.03158

**Published:** 2019-01-18

**Authors:** Samira D. Resende, Fernanda C. Magalhães, Jailza L. Rodrigues-Oliveira, Vanessa N. Castro, Carolina S. A. Souza, Edward J. Oliveira, Mariângela Carneiro, Stefan M. Geiger, Deborah A. Negrão-Corrêa

**Affiliations:** ^1^Department of Parasitology, Federal University of Minas Gerais (UFMG), Belo Horizonte, Brazil; ^2^Schistosomiasis Laboratory, René Rachou Research Center, Oswaldo Cruz Foundation, Belo Horizonte, Brazil

**Keywords:** *Schistosoma mansoni*, low parasite burden, humans, IgE-reactivity, household dust allergen, immunoregulation, IL-10, IL-33

## Abstract

Helminth infections and allergies are characterized by a predominant type-2 immune response. In schistosomiasis, the Th-2 response is usually accompanied by induction of immunoregulatory mechanisms that contribute to worm survival and less severe schistosomiasis. Although helminth-induced immunomodulatory mechanisms seem to affect atopy, epidemiological studies on the relationship between helminths and allergy have been inconsistent, and data suggest that the modulatory effects may be influenced by helminth species, chronicity of infection, and parasite burden. Here we performed a cross-sectional study to investigate the effects of *Schistosoma mansoni* parasite burden and immune response on allergic reactivity of individuals living in a schistosomiasis endemic area in Brazil. Fecal samples from the participants were collected for extensive parasitological examinations by spontaneous sedimentation, Kato-Katz, Helmintex and Saline Gradient tests and molecular detection of *S. mansoni* by qPCR. Additionally, the concentrations of cytokines and chemokines, total IgE and IgE-reactivity to common house dust allergens were quantified from serum samples. IgE reactivity to dust allergens was detected in 47 individuals (23.8%), and 140 individuals (54.4%) were diagnosed with *S. mansoni* infection. Most of the infected population (108 individuals) presented very low parasite burden (≤12 eggs/g of feces). The frequency and intensity (*p* ≤ 0.03) of allergic reactivity were lower in *S. mansoni-*infected compared with non-infected individuals. Multivariable logistic regression models adjusted by age revealed that allergic reactivity was positively associated with low IL-10 response (OR, 4.55, 95% CI, 0.56–7.36) and high concentration of the inflammatory mediators IL-33 (OR, 2.70, 95% CI, 1.02–7.15) or TNF-α (OR, 6.88, 95% CI, 0.32–143.39) in serum, and inversely associated with *S. mansoni* infection (OR, 0.38, 95% CI, 0.16–0.87). Most importantly, the logistic regression demonstrated that the modulatory effects of *Schistosoma* infection depend on parasite burden, with individuals infected with ≤12 eggs/g of feces showing allergic IgE-reactivity similar to non-infected individuals Altogether, our data show that immunomodulation of allergic reactivity depends on *S. mansoni* burden, low type-2 inflammatory response, and high level of IL-10.

## Introduction

Allergies are chronic diseases characterized by an intense and uncontrolled type-2 inflammation, with increased expression of typical cytokines such as interleukin (IL)-4, IL-5, IL-9, IL-13, eosinophilia and mast cell activation, elevated reactive immunoglobulin (Ig)E production, and increased mucus production in response to allergen exposition (1–3). Recent experimental work demonstrated the essential role of epidermal barrier integrity (4–7) and associated microbiota (8–11) in the production of innate cytokines/alarmins capable of interfering with dendritic cell maturation and innate lymphocyte subset activation, which are essential for differentiation and modulation of Th-2 responses (12).

Allergic inflammation results from a combination of genetic predisposition and environmental exposure and may affect different body parts resulting in clinical manifestations such as asthma, eczema, rhinitis, hay fever, and food allergies (12–14). The prevalence and the severity of these allergic manifestations highly increased among the human population after the second half of the twentieth century, especially among people living in urban areas of industrialized countries (15, 16). The genetic predisposition is, by itself, insufficient to explain the fast and heterogeneous increase of allergies in the human population, suggesting that environmental factors such as air pollution, diet, and exposition to infectious diseases could have an important role in increasing the risk of allergy (17–20). Data from experimental models (21–23) and epidemiological studies (24–27) suggest an inverse association between allergies and helminthic infections.

Although helminth infections are characterized by predominant type-2 immune response, they also stimulate an immune regulatory network response with production of anti-inflammatory cytokines, IL-10 and TGF-β, which modulate the immunopathology and facilitate parasite survival (28–31). Previous experimental data indicate that the regulatory network induced by helminths may also modulate other inflammatory process and, consequently, reduce the severity of inflammatory diseases, such as allergies (19, 32–34).

However, the inverse association between allergies and helminth infection is still unclear. A recent cross-sectional survey performed in fishing communities from Uganda with a high prevalence of intestinal schistosomiasis and soil-transmitted helminthiasis showed strong evidence that individuals with certain helminth infections, especially *Schistosoma mansoni* and *Trichuris trichiura*, were more prone to atopy (35). On the other hand, a systematic review and meta-analysis including 33 epidemiological studies demonstrated the lack of a significant effect of nematode parasite infection on the risk of developing asthma (36). In species-specific analysis, this same study showed that *Ascaris lumbricoides* infection was associated with an increased risk of asthma, while hookworm infection led to a significant reduction of asthma, in a burden-dependent manner (36). Additionally, Hunninghake et al. (37) observed that sensitization to *A. lumbricoides* was associated with increased severity and morbidity of asthma among children in Costa Rica (37). A suggested explanation for the positive association between *Ascaris* or *Toxocara* infection and asthma risk is the presence of high degree of immunological cross-reactivity between worm antigens and the environmental allergens that could increase Th-2 induction (38).

Therefore, the effect of helminth infection on allergic disease is complex and not entirely understood, and multiple factors, including helminth species and time of host infection, parasite burden, site and chronicity of the infection, are determinants in the modulatory outcome (39–42). Most of the experimental studies evaluated the modulatory effect of helminth infection as a preventive strategy to control chronic inflammation (43–45), while clinical trials (46, 47) designed to evaluate the therapeutic effect of helminth infection on chronically established allergy did not show considerable improvements, indicating the need for further studies. Another important aspect to be considered, is that the majority of the epidemiological studies with human subjects showing an inverse association between helminth infection and allergies were performed with chronically exposed human populations with high parasite burden.

In recent years, however, the improvement of sanitation and medical assistance in urban areas of developing countries and the multiple efforts to control helminth infections, including schistosomiasis, have led to a reduction in parasite burden in many areas around the world (47–49). Nevertheless, the effect of this new epidemiological scenario of schistosomiasis on the induction of modulatory response has not yet been evaluated. To better understand the impact of schistosomiasis infection on the development of allergic diseases in this new context, we evaluated the relationship between *S. mansoni* infection and the circulating levels of immune mediators and the IgE-reactivity to common household dust allergens in individuals from a rural community of a schistosomiasis endemic area in Brazil. Our data showed that *Schistosoma* infection can reduce the prevalence and intensity of allergic reactivity to common household dust allergens and, most importantly, that helminth-induced modulation is dependent on parasite burden.

## Materials and Methods

### Ethics Statement

The present study was approved by the Ethics Committee of the Research Center René Rachou—FIOCRUZ (Belo Horizonte, MG–Brazil) and all project details have been registered on the Brazilian Platform for Research with Human Subjects (Plataforma Brasil—protocol number: CAAE#21824513.9.0000.5091). Prior to the commencement of the research activities, the subjects were invited to participate in local meetings or receive house-to-house visits to hear about the aims of the research and any possible risks. All enrolled participants and/or their legal guardians agreed to participate in the research and sign an informed consent form. All the data were anonymized prior to analysis. After the evaluations, the individual test results were sent to each participant and, regardless of participation in the study, patients with confirmed parasite infection received treatment and patients with other diseases were either treated at the local health clinic or directed to specialized treatment. Schistosomiasis cases were treated with praziquantel (adults: 40 mg/kg; and children: 60 mg/kg); intestinal helminthiases were treated with albendazole (400 mg); and protozoan parasites were treated with metronidazole (250 mg/2x/5 days).

### Study Population and Experimental Design

The current study was conducted in 2014 with individuals from a rural community near the village of Brejo do Amparo, Municipality of Januária, located in the northern area of the state of Minas Gerais, Brazil (15° 29′ 16″ S 44° 21′ 43″ O). This rural community is historically endemic for schistosomiasis and currently presents around 270 residents. According to the Brazilian Schistosomiasis Control Program and local health authorities, the estimated prevalence of schistosomiasis was around 20% in 2010 (50), and no control interventions had been put into place in this locality in the 2 years prior to the present study.

**Figure 1:**
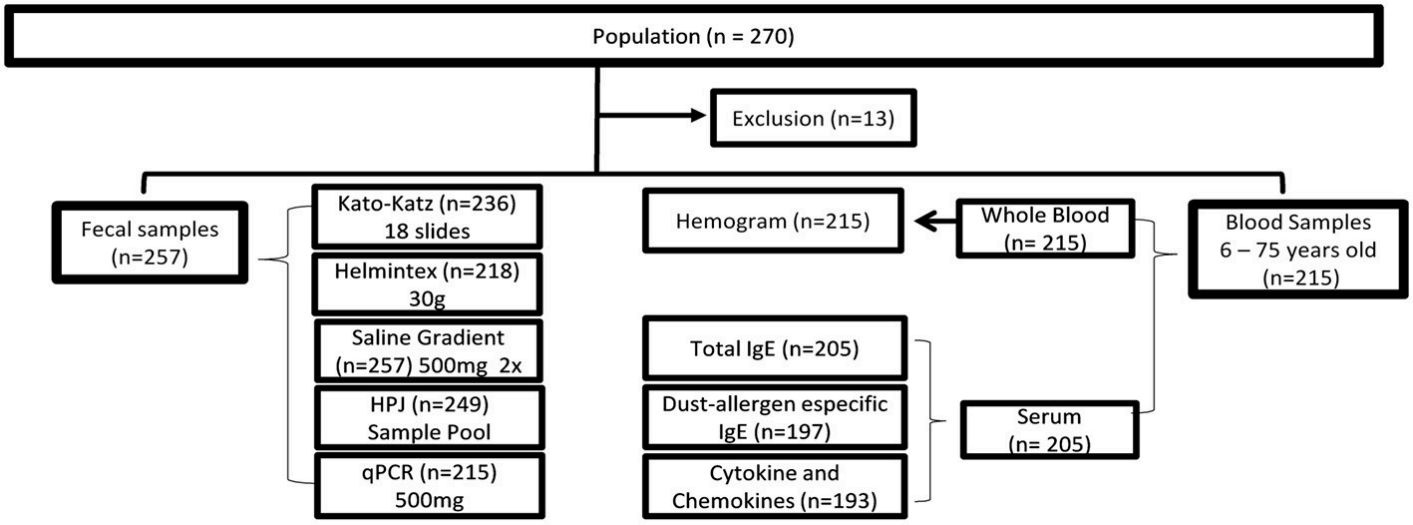
Flowchart describing the cross-sectional study within the district of Brejo do Amparo population, Januária, Minas Gerais, Brazil. Of the 270 individuals residing in the region, 257 signed the consent form and had stool samples collected for analysis by a combination of parasitological and molecular tests: Kato-Katz, Helmintex, Gradient Saline, spontaneous sedimentation test (HPJ), and qPCR. Blood samples were collected from 215 subjects aging from 6 to 75 years old. Whole blood samples were submitted to a complete blood count while serum samples were used for immunological tests: Total IgE, Dust-allergen specific IgE, and quantification of cytokines and chemokines. The number of samples used in each of the tests is indicated between parentheses. HPJ: Hoffman, Pons, and Janner.

We carried out a cross-sectional population-based study including residents of both sexes, aging between 2 and 88 years old who signed the informed consent form. Pregnant women and individuals who could not understand and/or cooperate with the study protocol were excluded. The participants were asked to provide three fecal samples, which were collected on consecutive days and used for the parasitological analysis by different methods. The first fecal sample, containing at least 50 grams of feces, was collected in a 500-ml plastic container and used for complete fecal evacuation. The other two fecal samples were smaller and were collected in 80-ml plastic pots. Participants aging between 6 and 75 years old were also invited to donate a blood sample, which was used for immunological analysis. Five Milliliter of venous blood were collected from each participant in Ethylenediaminetetraacetic acid (EDTA)-coated tubes (Biocon, Belo Horizonte, Brazil). Hemogram analyses were performed by a clinical laboratory located in the region and peripheral blood eosinophil counts were used in current study. Additionally, 10–15 ml of blood were collected without anticoagulant, and serum samples were aliquoted and stored at −20°C for subsequent quantification of circulating cytokine and chemokines, total IgE, and the level of IgE reactivity to common domestic dust allergens. The flow diagram in Figure 1 illustrates the study design and the total number of samples analyzed by each method.

The participants or their legal guardians answered an individual questionnaire providing demographic and occupational information, as well as previous clinical conditions that could be relevant for the research. A family-based questionnaire was also applied to gather information on household construction, water supply, sanitation, and other socio-economical aspects.

### Parasitological Analysis

The parasitological analysis adopted herein has been previously described (51). Briefly, 500 mg of feces from the first fecal sample were separated and immediately frozen for molecular diagnostic test. The remaining fecal material from the first stool sample was used to prepare 14 Kato-Katz slides (52). Additionally, 500 mg of feces were used to perform the Saline Gradient technique (47), 30 g were used for the Helmintex® technique (53), and the remaining sample was processed for the spontaneous sedimentation method (54). The second and third stool samples were used to prepare two Kato-Katz slides, thus totalizing 18 Kato-Katz slides per individual. The slides containing fecal material from the different parasitological tests were examined under the microscope by trained technicians to evaluate the presence of helminth eggs or larvae and protozoan cysts.

The clinical record of cutaneous leishmaniasis, diagnosed and documented by the local health program, was included in the analysis since this protozoan disease is also endemic among the study population.

### DNA Extraction and qPCR

Total DNA was extracted from 500 mg stool samples using the commercial QIAamp® DNA Stool Mini Kit, following the manufacturer's instructions (Qiagen GmbH, Hilden, Germany). The primers sense 5′-CCG ACC AAC CGT TCT ATG A-3′ and anti-sense 5′-CAC GCT CTC GCA AAT AAT CTA AA-3′ and the probe 5′-6[FAM]/TCG TTG TAT CTC CGA AAC CAC TGG ACG/[(3BHQ1)] (Integrated DNA Technologies—IDT-USA) were used in the PCR reaction, as previously described (55). As reported before (56), these primers and probe sets amplify and detect a 90 bp fragment of a highly repetitive 121 bp sequence of *S. mansoni* (GenBank, accession number M61098). The amplification reaction was performed in a final volume of 25 μl containing: 12.5 μl of TaqMan® Universal PCR Master Mix (Life Technologies, Thermo Fisher Scientific Inc., USA), 0.1 μM of each *S. mansoni*-specific primer, 0.25 μM of probe, BSA 0.1 μg/μL; 4 mM of MgCl_2_; and 4 μl of 5-fold diluted stool DNA sample. For each run, positive (DNA extracted from adult worms) and negative (no DNA template) controls were performed. The amplification reaction was conducted on the StepOnePlus™ Real-Time PCR System (Thermo Fisher Scientific Inc., USA) using the universal cycling program with 45 cycles and annealing temperature of 60°C. A 92 pb fragment of the human β-actin gene was amplified and detected as an internal control. The cut-off for positive and negative samples was defined by a standard curve established with *S. mansoni* DNA extracted from adult worms. The DNA extraction and amplification procedures were performed by a trained researcher in separate rooms inside a biological safety cabinet using disposable sterile pipette tips with filters.

### Diagnosis of Intestinal Schistosomiasis and Determination of Parasite Burden

Individuals who presented eggs in any of the parasitological tests or positive PCR-reaction were considered positive. The intensity of infection was calculated by determining the mean number of *S. mansoni* eggs detected in six slides of Kato-Katz (two slides from each of the three fecal samples) and multiplying the mean value obtained by 24 to determine the number of eggs per gram of feces (EPG). Individuals who had a positive result for *S. mansoni* infection in qualitative tests (Helmintex®, Saline Gradient and/or qPCR), but were negative in the six Kato-Katz slides were classified as infected with parasite burden < 4 EPG.

### Serum Concentration of Total IgE

The total IgE concentration in each serum sample was determined by a commercial kit (Bethyl, Montgomery, USA) following the manufacturer's instructions and a previously optimized protocol (57). Serum samples were diluted 1:100 in Tris-NaCl buffer containing 0.1% of bovine serum albumin (BSA-Sigma) and tested in duplicates. The reaction was developed using Tetramethyl-benzidine (TMB) substrate solution (R&D Systems, Minneapolis, USA). After 30 min, the reaction was stopped with 100 μL of 4NH_2_SO_4_ solution, and absorbance was determined using a 450 nm filter in the ELISA reader (VersaMax, Molecular Devices, Sunnyvale, CA). Known concentrations of the recombinant human IgE were used to generate a standard curve, which was used to determine the IgE concentration (ng/mL) in each serum sample based on the optical density (OD) readings.

### IgE Reactivity to Household Dust Allergens

The serum samples were additionally used for quantification of IgE reactivity against common household dust antigens using the commercially available IgE REAST kit (Dr. Fooke, Neuss, Germany) and following the manufacturer's recommendations. For the current analysis, we selected the HMx3 allergenic mixture composed of the common household dust allergens, including *Dermatophagoides pteronyssinus, D. farinae, Cladosporium herbarum, Aspergillus fumigatus*, and cat and dog fur antigens. Undiluted serum samples from the study population, positive and negative serum controls, and samples containing known concentrations of allergen-reactive IgE for the standard curve were added to the anti-human IgE-sensitized microplates provided by the fabricant. Following washing, 100 μl of the biotin-conjugated allergen mixture (HMx3) was added to the wells containing the serum samples and 100 μl of biotin-conjugated anti-IgE antibody solution was added to the wells with the standard curve. This was followed by the addition of peroxidase (HRP)-conjugated streptavidin solution. The reaction was developed with 100 μl/well of the substrate solution containing Tetra-methyl-benzidine (TMB) and stopped after 20 min by the addition of 100 μl of 4NH_2_SO_4_ solution. The color intensity was measured spectrophotometrically at 450 nm (Molecular Devices—Versa Max). The concentrations of reactive-IgE in serum from each patient were estimated based on the reactivity of the standard curve and expressed as International Units (IU). All patients with reactivity above 0.35 IU/ml were considered reactive. The intensity of IgE-reactivity was expressed as total concentration or categorized as Non-reactive (<35 IU/ml), Low (0.35–0.7 IU/ml), Moderate (0.7–17.5 Ul/ml), or High (>17.5 IU/ml).

### Cytokine and Chemokine Analysis

Concentrations of pro-inflammatory cytokines, such as IL-1β, IL-6, TNF-α, chemokines and cytokines of type1/17, IL-27, CXCL-10, CCL-3, IL-17, regulatory and type-2, IL-10, IL-5, IL-13, IL-33, CCL-5, CCL-11, CCL-17 were measured in duplicate in serum samples from each participant using a sandwich enzyme-linked immunosorbent assay (ELISA) kit, according to the manufacturer's instructions (Duoset, R&D Systems, EUA). Each serum sample was diluted in phosphate buffer (1:2) with 0.1% BSA (PBS/BSA), except for CCL-5, which was diluted 1:20 in PBS/BSA solution. Known concentrations of the recombinant proteins were used to generate a standard curve to determine the concentration (pg/ml) of the samples based on OD readings.

### Processing and Analysis of Data

Categorical variables were compared using the *x*^2^ test, means were compared using Student's *t*-test or analysis of variance (ANOVA), and the Kruskal-Wallis test was used to compare medians. Correlation analysis was used to quantify the association between continuous variables. Logistic regression models were used to assess the relationship between IgE-reactivity against common house dust allergens (allergic reactive and non-reactive individuals) and the variables analyzed (demographics, *Schistosoma* infection and immune mediators). The strength of the association was assessed using the odds ratio (OR) with a 95% confidence interval (CI). Variables with *p*-values < 0.25 in the univariate analysis were selected for construction of the multivariate logistic regression models. Variables with low frequency and showing co-linearity were excluded from the multivariate analysis. For the categorical variables, the categories non-responder or non-infected were used as reference values for the logistic regression. The likelihood ratio test was used to define the final model with the best data fit (58).

## Results

### Characterization of the Study Population

At the time of data collection, the rural community of Brejo do Amparo had approximately 270 residents. Among those, 257 individuals, from 53 families, were eligible to participate in the study. Forty-nine percent were males and 51% females, and the age ranged from 2 to 88 years old with a median age of 32 years (interquartile range 15–51 years), equally distributed throughout the age groups (Table 1). Most of the individuals (82%) declared having frequent contact with the river water and soil, which are the main sources of parasite contamination. Moreover, the rural community had no access to treated water or sewage treatment; most residences used the local river as a source of drinking water (60.3%), and most of the sewage was disposed of in rudimentary cesspools (88.6%). Almost 80% of the participants declared having no formal education or only basic educational level and more than 60% of them earned minimal Brazilian wages or less (Table 1).

**Table 1:** Demographic and socio-economic characterization of individuals living in the rural community of Brejo do Amparo, Januária MG, Brazil.

**Variables**	**Category**	**Number (%)**
Gender^a^	Male	112 (48.9)
	Female	117 (51.1)
Age Group^a^	≤10	38 (16.6)
	11–20	43 (18.8)
	21–40	61 (26.6)
	41–60	60 (26.2)
	>60	27 (11.8)
Education level^a^	No education	123 (59.4)
	Primary school	33 (15.9)
	Secondary school	43 (20.8)
	Higher education	8 (3.9)
Income^b^	<1 minimum wage	19 (35.9)
	1–2 minimum wages	16 (30.2)
	>2 minimum wages	18 (34.0)
Water supply^c^	Covered well	21 (39.6)
	Stream	32 (60.4)
Sewage Disposal^c^	Rudimentary cesspool	47 (88.6)
	Does not know or no answer	6 (11.4)

a *Variables evaluated in the individual questionnaire (229 residents)*.

b *Variable evaluated in the individual questionnaire excluding children under 6 years of age (207 residents)*.

c *Variables evaluated in the family questionnaire (53 residences)*.

Among the participants, 58 individuals had no detectable intestinal parasite infection. Fecal examination by Spontaneous Sedimentation revealed four individuals eliminating *Giardia lamblia* cysts, nine with *Entamoeba histolytica/dispar* cysts and 72 individuals with commensal protozoan cysts in their feces (Table 2). To ensure a precise diagnosis of intestinal schistosomiasis, multiple parasitological methods for fecal examination (Kato-Katz, Saline Gradient, Helmintex) and a molecular method (qPCR) were applied. The combined methods identified 140 (54.4%) individuals with *S. mansoni* infection, including 59 that had only *S. mansoni* infection and 81 that were co-infected. The analysis also identified 23 individuals eliminating eggs of hookworms and 6 individuals with *Enterobius vermicularis*, and the majority of these nematode infected individuals were co-infected with another parasite infection (Table 2). Interestingly, 12 of the 19 hookworm co-infected and 4 of the 5 *E. vermicularis* infected individuals were co-infected with *S. mansoni*. There was also one case of *Trichuris trichiura* and one of *Strongyloides stercoralis* infection. Eighty (35%) participants reported past cutaneous leishmaniasis infection (Table 2).

**Table 2:** Parasite infection status established by clinical examination and a combination of parasitological and molecular diagnostic tests in residents of the rural community of Brejo do Amparo, Januária MG, Brazil.

**Parasite infection status**	**Mono-infected^**a**^**	**Co-Infected^**b**^**	**Total *n*(%)**
Non-infected	–	–	58 (23%)
*Leishmania* sp.	19	61	80 (31%)
Comensal protozoa^c^	11	61	72 (28%)
*Giardia lamblia*	1	3	4 (2%)
*Entamoeba histolytica/díspar*	0	9	9 (4%)
*Schistosoma mansoni*	59	81	140 (54%)
Ancilostomídeo	4	19	23 (9%)
*Enterobius vermicularis*	1	5	6 (2%)
*Strongyloides stercoralis*	1	0	1 (0.5%)
*Trichuris trichiura*	1	0	1 (0.5%)

a *Individuals with only 1 parasite detected*.

b *Individuals with 2 or more parasites detected*.

c *Cysts of Entamoeba coli, Endolimax nana, Iodamoeba buetschli, and/or Blastocystis hominis*.

### *Schistosoma mansoni* Infection and IgE-Reactivity to Dust Allergens

**Figure 2:**
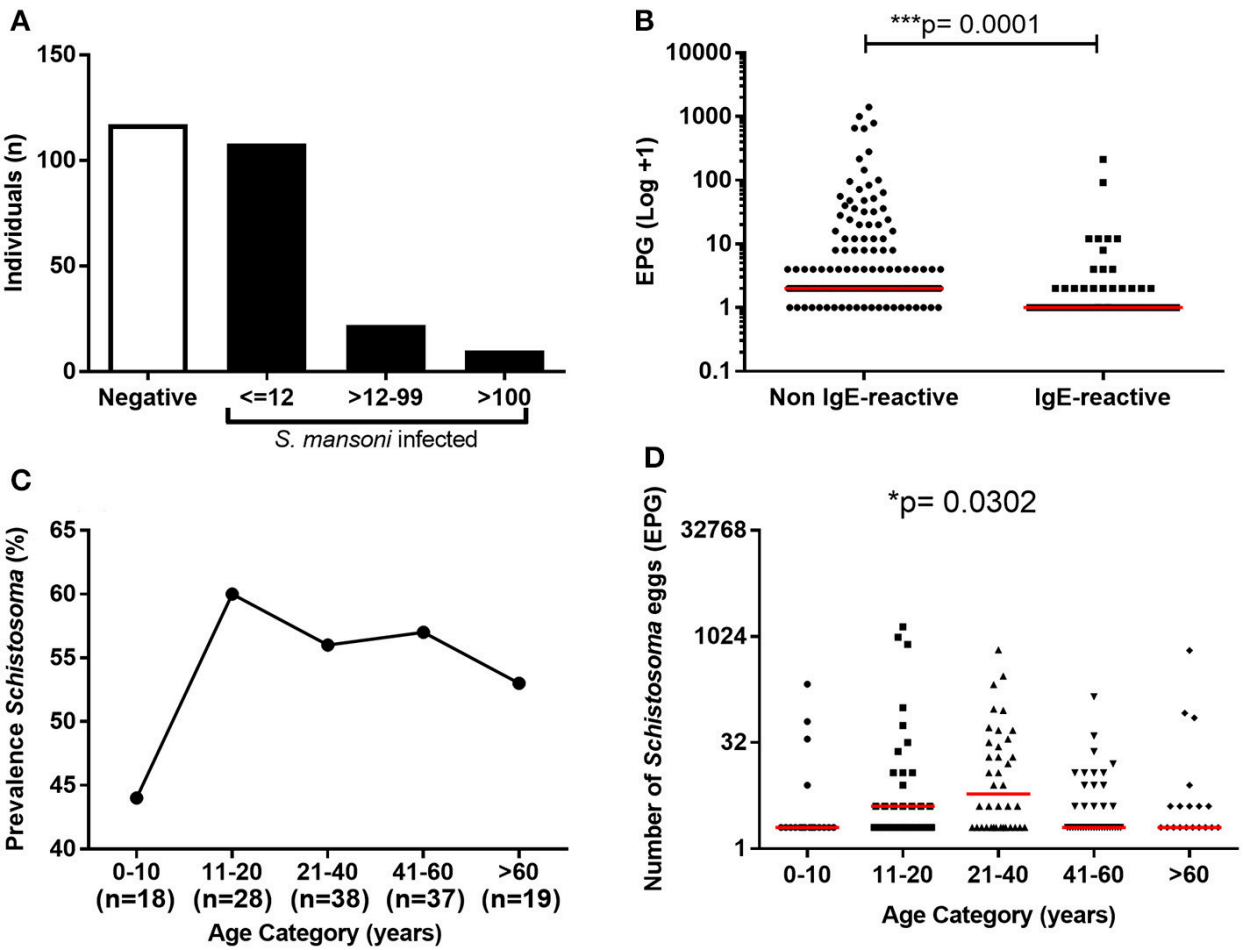
Prevalence and parasite load of *S. mansoni* infection among the study population. **(A)** Frequency of *S. mansoni* infection and parasite load among the individuals; **(B)** Parasite load in IgE-reactive and non-reactive individuals. **(C)** Prevalence of *S. mansoni*-infection by age range; **(D)** Parasite load (EPG) by age range. In B and D the points represent number of eggs eliminated by each individual and the horizontal bars the median values. Comparison between the groups were done by Mann-Whitney or Kruskal-Wallis test for multiple comparison and the *p*-value of the comparison was assigned.

The parasite burden in *S. mansoni*-infected individuals based on egg counts from six Kato-Katz slides from three fecal samples revealed that only 10 individuals (7%) presented with moderate or high parasite burdens (>100 EPG). The remaining 130 infected individual (93%) showed low parasite burden (≤100 EPG). Noteworthy, among the individuals with low parasite burden, the vast majority (83%) eliminated less than 12 EPG (Figure 2A). The schistosomiasis prevalence in individuals under 10 years old was 44%, while 60% of individuals between 11 and 20 years old, and 53–56% of individuals in the older age groups presented the infection (Figure 2C). Although most of the infected individuals had a low number of eggs in feces, the parasite burden was slightly higher in individuals between 11 and 40 years old (Figure 2D). Moreover, *S. mansoni* parasite burden was significantly lower in IgE-reactive individuals than in non-IgE-reactive (Figure 2B).

**Figure 3:**
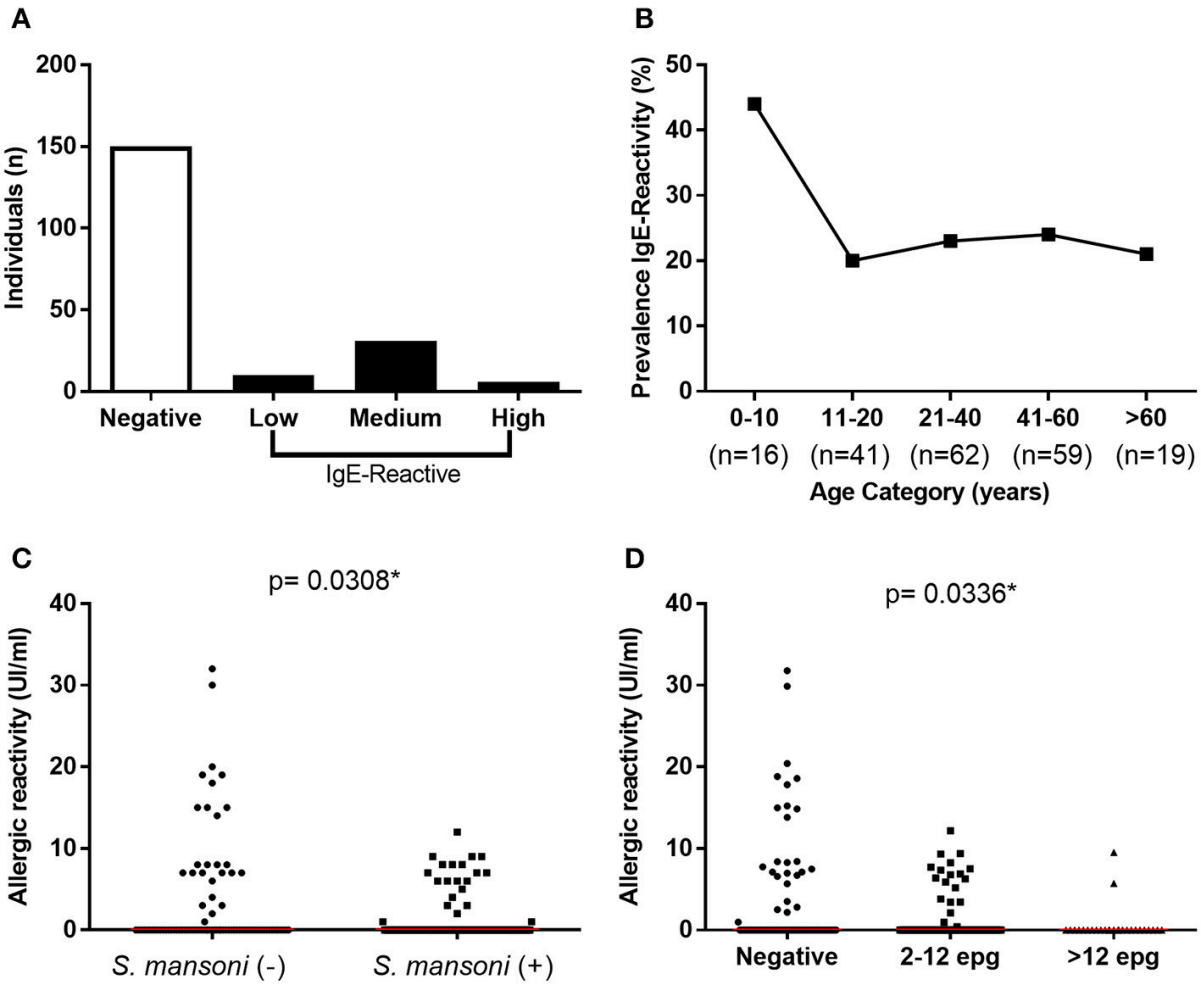
Prevalence and intensity of IgE-reactivity against dust mite household allergens among the study population. **(A)** Frequency and intensity of IgE reactivity among the individuals; **(B)** Prevalence of IgE reactivity by age range. **(C)** Intensity of IgE-reactivity among *S. mansoni*-infected and non-infected individuals; **(D)** Intensity of IgE-reactivity by parasite load. In **(C,D)** the points represent the intensity of IgE-reactivity by each individual and the horizontal bars the median values. Comparison between the groups were done by Mann-Whitney or Kruskal-Wallis test for multiple comparison and the *p*-value of the comparison was assigned.

Of the 197 individuals tested for IgE-reactivity against dust allergens, 47 (23.9%) were reactive (≥0.35 IU/mL). The median intensity of IgE-reactivity in this population was 7 IU/mL (IQR 5.1–9.5), and six individuals showed strong reactivity (≥17 IU/mL) (Figure 3A). The rate of IgE-reactivity was 44% among children under 10 years old and remained between 21 and 24% in older individuals (Figure 3B); however, there was no statistical difference in the intensity of IgE-reactivity to dust allergens among the different age groups.

It is important to mention that among the 47 IgE-reactive individuals, 44.7% presented *S. mansoni* infection, while up to 60% of non-IgE-reactive individuals (150 individuals) were infected with the parasitic trematode (*p* = 0.06). Moreover, the intensity of IgE-reactivity against dust allergens was stronger in non-infected individuals in comparison with *S. mansoni*-infected individuals (*p* = 0.03) (Figure 3C). Most importantly, a significant reduction of IgE-reactivity to dust allergens was detected only in infected individuals that eliminated more than 12 EPG (Figure 3D).

### Immune Response Profile

**Figure 4:**
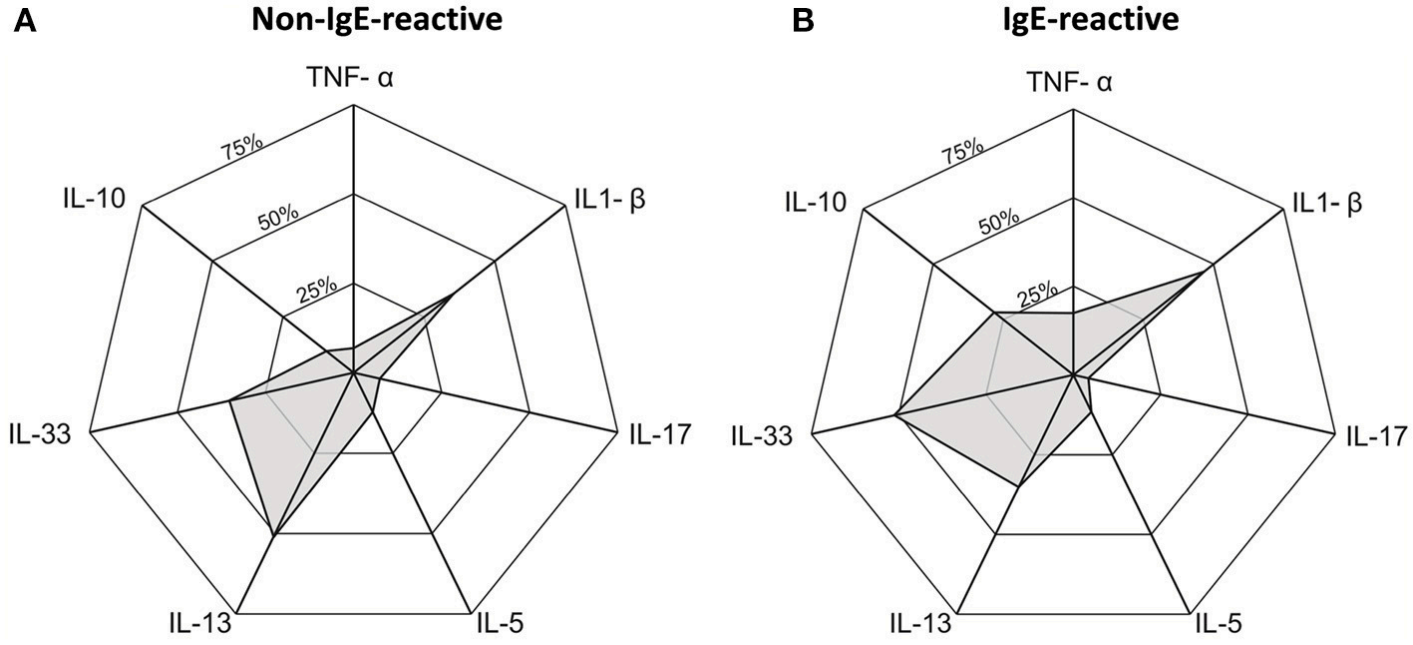
Radar graph showing the frequency of cytokine responder in serum of individuals non-IgE-reactive **(A)** and IgE-reactive **(B)** to common dust allergens in a rural area endemic for intestinal schistosomiasis.

Most of the individuals evaluated in the current study presented undetectable serum levels of TNF-α, IL-1β, IL-5, IL-10, IL-13, IL-17, and IL-33. Therefore, we compared the frequency of individuals with a detectable level of these cytokines in serum (cytokine-responders) among IgE-reactive and non-IgE-reactive individuals, independently of the parasitological status. As demonstrated in Table 3, the frequency of responders for IL-1β, IL-5, and IL-17 was low and comparable among individuals with and without IgE-reactivity to dust allergen. In contrast, the frequency of TNF-α (*p* = 0.031) and IL-10 (*p* = 0.001) responders was significantly higher in the IgE-reactive group in comparison with the non-IgE-reactive individuals. A higher percentage of IgE-reactive individuals showed detectable IL-33 (*p* = 0.071), while a higher percentage of IL-13 responders was observed in the non-IgE-reactive population (*p* = 0.085) (Table 3). Figure 4 illustrates the frequencies of cytokine responders in IgE-reactive vs. non-IgE-reactive individuals.

**Table 3:** Frequency (n and %) of detectable cytokine responses in the serum of individuals with non-reactive and reactive IgE concentrations against common house dust allergens.

**Cytokine**	**Non-reactive for specific IgE**	**Reactive for specific IgE**	***p* value^**a**^**
	**150 (76.1%)**	**47 (23.8%)**	
**IL1-β** **(*****n*** **=** **192)**			0.172
Undetectable	95 (64.6%)	24 (53.3%)	
Detectable	52 (35.4%)	21 (46.7%)	
**IL-6 (*****n*** **=** **192)**			0.176
Undetectable	42 (28.4%)	8 (18.2%)	
Detectable	106 (71.6%)	36 (81.8%)	
**TNF-α** **(*****n*** **=** **193)**			0.031^*^
Undetectable	137 (93.2%)	38 (82.6%)	
Detectable	10 (6.8%)	8 (17.4%)	
**IL-17 (*****n*** **=** **193)**			0.459
Undetectable	136 (92.5%)	44 (95.7%)	
Detectable	11 (7.5%)	2 (4.3%)	
**IL-10 (*****n*** **=** **192)**			0.001^***^
Undetectable	132 (90.4%)	33 (71.7%)	
Detectable	14 (9.6%)	13 (28.3%)	
**IL-5 (*****n*** **=** **192)**			0.919
Undetectable	115 (87.8%)	38 (88.4%)	
Detectable	16 (12.2%)	5 (11.6%)	
**IL-13 (*****n*** **=** **168)**			0.085
Undetectable	64 (48.9%)	24 (64.9%)	
Detectable	67 (51.1%)	13 (35.1%)	
**IL-33 (*****n*** **=** **175)**			0.071
Undetectable	88 (64.7%)	19 (48.7%)	
Detectable	48 (35.3%)	20 (51.3%)	

a *Chi-quadrade test (x^2^)*,

* p ≤ 0.05 and

*** *p ≤ 0.001*.

In contrast, almost all the individuals in the study population showed detectable serum levels of the chemokines CCL-3, CCL-5, CXCL-10, CCL-11, and CCL-17, and the cytokine IL-27. Table 4 shows the median values of these immune mediators for the IgE-reactive and non-IgE-reactive individuals. The concentrations of these mediators were statistically similar in both groups (Table 4).

**Table 4:** Cytokine concentration (pg/ml) in the serum of individuals with IgE-reactivity against common house dust allergens and non-IgE-reactive individuals of the study population.

	**Non-reactive for specific IgE**		**Reactive for specific IgE**	
**Cytokines/Chemokines (pg/mL)**	**Median**	**IQR (25–75%)**	**Median**	**IQR (25–75%)**	***p* value^**a**^**
IL-27	700	330–1,056	623	400–862	0.623
CXCL-10	111	72–173	120	90–178	0.248
CCL-3	5,970	3,360–1,555	5,360	2,900–309	0.943
CCL-5	11,695	7,498–21,857	13,245	6,328–32,397	0.602
CCL-11	74	24–181	91	24–192	0.497
CCL-17	251	109–1,079	228	117–688	0.735

a *Mann-Whitney test. IQR, Interquartile range*.

**Figure 5:**
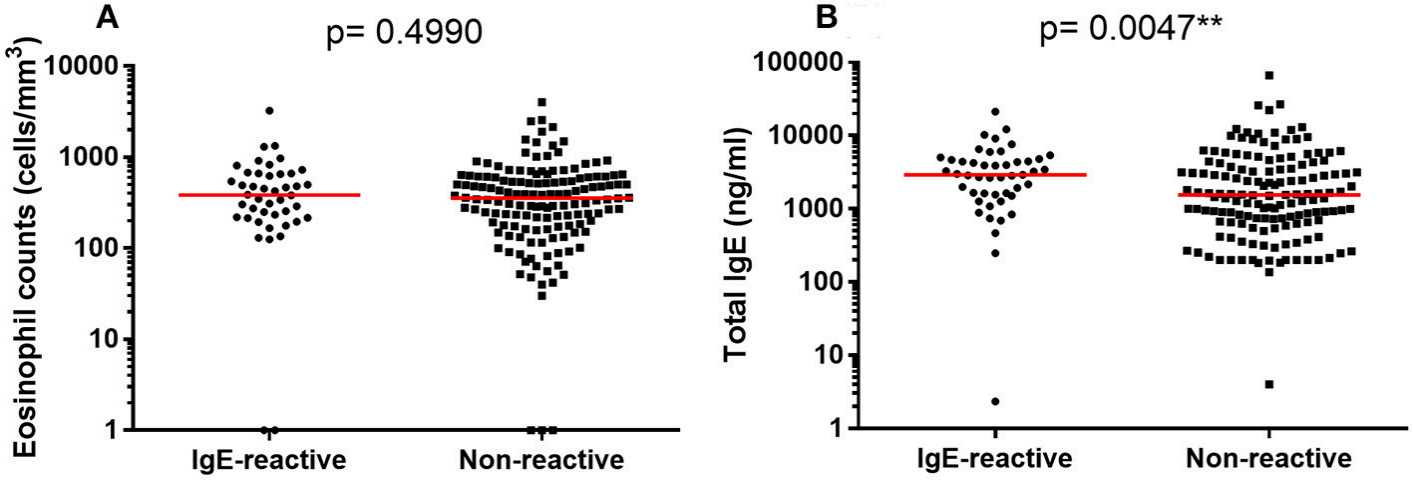
Number of circulating eosinophils and total IgE concentration in serum of allergic-reactivity in individuals a rural area endemic for intestinal schistosomiasis. **(A)** Number of circulating eosinophils in dust-allergen IgE-reactive and non-IgE-reactive individuals. **(B)** Serum concentration of total IgE in dust-allergen IgE-reactive and non-IgE-reactive individuals. The median values are represented as horizontal bars. Comparison between the groups were done by Mann-Whitney test for multiple comparison and the *p*-value of the comparison was assigned.

We also evaluated the total IgE concentration in serum and the number of circulating eosinophils (Figure 5). The median values of circulating eosinophils were similar in IgE-reactive and non-IgE-reactive individuals (Figure 5A). However, the total IgE concentration was significantly higher in IgE-reactive compared with non-IgE-reactive individuals (Figure 5B). Spearman correlations of total IgE and eosinophils with parasite burden (EPG) and with intensity of IgE-reactivity revealed no statistically significant associations (data not shown).

### Multivariable Regression Models

Tables 5, 6 show the final logistic regression models that describe the effect of different immune mediators and *Schistosoma* infection status on IgE-reactivity against common house dust allergens. The first model was built using the presence of *Schistosoma* infection (mono- and co-infected) or absence of infection (negatives and any other parasitic infection) and it was adjusted by individual age. The analysis showed that allergic reactivity was inversely associated with the presence of infection and positively associated with low (≤100 pg/ml) serum concentration of IL-10 and high (>100 pg/ml) concentration of IL-33. Although CXCL-10 was statistically associated with IgE-reactivity (Table 5), it presented OR close to 1 (OR, 1.0031, 95%, CI = 1.0006–1.0057), and the variable was maintained to better fit the model.

**Table 5:** Association between allergic reactivity against common house dust allergens, *Schistosoma mansoni* infection and serum immune mediators, by the final multivariable regression model analysis.

	**Allergic reactivity**			
**Variables**	**Odds ratio**	**Z-score**	***p*-value**	**CI 95%**
*S. mansoni* infection	0.38	−2.27	0.02^*^	0.16–0.87
IL-10 ≤ 100 pg/ml	4.82	2.36	0.01^*^	1.30–17.85
IL-10 > 100 pg/ml	1.48	0.49	0.62	0.30–7.36
IL-33 ≤ 100 pg/ml	1.40	0.59	0.55	0.44–4.43
IL-33 > 100 pg/ml	2.70	2.01	0.04^*^	1.02–7.15
CXCL-10	1.00	2.02	0.04^*^	1.00–1.05

*Reference for IL-10 and IL-33 was the undetectable category. ROC curve value = 0.7294*.

* *p ≤ 0.05*.

**Table 6:** Association between allergic reactivity to common house dust allergens, concentrations of immune mediators and *Schistosoma* parasite burden, by the final multivariable regression model analysis.

	**Allergic reactivity**			
**Variables**	**Odds ratio**	***z* score**	***p*-value**	**CI 95%**
*Schistosoma* infection EPG ≤ 12	0.69	−0.92	0.35	0.32–1.49
*Schistosoma* infection EPG > 12	0.17	−2.12	0.03^*^	0.03–0.87
TNF-α ≤ 10 pg/ml	1.63	0.49	0.62	1.10–13.22
TNF-α > 10 pg/ml	6.88	2.54	0.01^*^	0.32–143.39
IL-10 ≤ 100 pg/ml	4.55	2.41	0.01^*^	1.20–12.67
IL-10 > 100 pg/ml	2.37	1.34	0.18	0.56–7.36

*Reference for Schistosoma infection was the non-infected category. Reference for TNF-α and IL-10 was the undetectable category. ROC curve value = 0.7356*.

* *p ≤ 0.05*.

A second regression model was established following the categorization of *Schistosoma* infection into very low (≤12 EPG) or high (>12 EPG) parasite burden. The analysis demonstrated an inverse correlation between allergic reactivity and *Schistosoma* infection only in infected individuals eliminating more than 12 EPG. The risk of development of allergic-reactivity was six times lower in these individuals. On the other hand, the protective effect was not observed in infected individuals with very low parasite burden (≤12 EPG). Additionally, allergic reactivity was also positively associated with low (≤100 pg/ml) serum concentration of IL-10 and high (>10 pg/ml) concentration of TNF-α (Table 6).

## Discussion

Helminth parasites are potent inducers of regulatory mechanisms capable of reducing inflammatory processes and autoimmune diseases (19, 35). Induction of immunomodulatory mechanisms has been used to explain epidemiological data reporting an inverse association between exposure to helminth infections and human chronic inflammatory diseases, including allergic conditions (19, 59). However, a putative causal relationship between helminth infection and reduction of allergic diseases is mainly supported by data from experimental animal models, while evidence from human studies are still controversial and suggest that aspects such as helminth species, chronicity and site of infection, and parasite burden should be taken into consideration (45, 60, 61). Here, we conducted a cross-sectional study in a schistosomiasis endemic area where most infected individuals harbor a small number of parasites, with the aim of evaluating whether low parasite burden could affect the modulation of allergic reactivity against common household allergens. Our analysis revealed that *S. mansoni* infection has a modulatory effect on IgE-reactivity to common house dust allergens in individuals residing in areas with frequent exposure to this helminth infection. However, the modulatory effect was only observed in individuals with parasite burden above 12 EPG. The modulation of allergic reactivity was also accompanied by changes in the systemic immune response, including serum concentrations of IL-33, TNF-α, and IL-10. Altogether, our data suggest that factors associated with helminth species, parasite burden, and frequency of exposition should be further explored in order to better understand the mechanisms underlying the immunomodulatory response derived from helminth infections, which may provide novel tools for the prevention of allergic diseases.

The state of Minas Gerais represents one of the largest endemic areas for schistosomiasis in Brazil, with regions of poor social and sanitary conditions that favors parasite transmission (48, 62, 63). This is the case of the rural community of Brejo do Amparo in the municipality of Januária, which was the focus of the current study. *S. mansoni* transmission has been historically reported in this endemic area, and the main control strategy consists on the treatment of infected individuals, which has contributed to the significant reduction of parasite infection intensity and, consequently, reduced schistosomiasis severity. Nevertheless, these control measures were insufficient to eliminate *Schistosoma*-transmission, thus resulting in infected individuals with low parasite burden, which hinders diagnosis of schistosomiasis, as reported in many schistosomiasis endemic areas (47, 48, 64).

Population-based studies evaluating the relationship between the parasitological status and allergic reactivity of individuals residing in such areas would help to understand the role of frequent exposition to infection and low parasite burden on induction of modulatory mechanisms that affect allergic responses. In the current study, we used a combination of different parasitological and molecular diagnostic methodologies to detect *Schistosoma*-infected individuals with very low parasite burden and identified *S. mansoni* infection in 54.4% of the studied population. The observed prevalence is much higher than the ~20% estimated by local health authorities (50). This might be explained by the comprehensive diagnostic procedures used herein, which allowed us to identify 108 infected individuals with very low parasite load (51), which would not be diagnosed by the Kato-Katz method only.

We observed that 47 (23.9%) individuals showed IgE-reactivity to a combination of common household dust allergens, which is similar to the prevalence of atopy observed in other areas of helminth transmission in rural communities or poor neighborhoods of Latin America. In the Province of Esmeraldas, Ecuador, it was reported that 26.5% of the population from an urban area and 10.5% of the individuals from rural communities were IgE-reactive (65). In Brazil, a large study with children living in poor neighborhoods of a city with a high prevalence of helminth infection revealed 37% of atopy, evaluated by allergen IgE-reactivity in serum (66, 67).

Our data also showed that the allergic reactivity was more frequent in non-infected than in *Schistosoma*-infected individuals, with the latter showing lower allergic reactivity intensity. Moreover, non-IgE-reactive individuals showed higher parasite load compared to IgE-reactive individuals, thus suggesting a modulatory effect of *S. mansoni* infection on the allergic process. The inverse association between allergic reactivity and *S. mansoni* infection was confirmed by the multivariable logistic regression model, which showed that individuals infected by *S. mansoni* were three times less likely to develop allergic reactivity to common dust allergens than non-infected individuals. Since schistosomiasis and allergies are chronic diseases, the regression model was adjusted by age, indicating that the effect of *Schistosoma* infection, as demonstrated here, was not dependent on host age. A similar outcome was observed for allergic reactivity intensity, with *S. mansoni*-infected individuals showing lower intensity of IgE-reactivity to dust allergens (data not shown).

This is in agreement with epidemiological studies that evaluated allergic reactivity using Skin Prick Test (SPT) or allergen-specific IgE (sIgE) in individuals living in helminth highly endemic areas and showed an inverse association between allergen reactivity and the presence of chronic helminth infections, such as *A. lumbricoides* and *T. trichiura* in rural areas of Ecuador (34), ancylostomiasis in Southeast Asia (68), and *S. haematobium* in children from Gabon (24). In case of *S. mansoni* infection, Medeiros et al. (69) demonstrated that asthmatic individuals living in areas with high prevalence of schistosomiasis showed milder course of asthma compared to asthmatic patients living in non-endemic areas (69). Moreover, meta-analysis data confirmed that *Schistosoma* infection reduced atopic risk (70). Altogether, these data show that the frequent exposure to helminthiasis may affect the local immunoregulatory responses that are capable of modulating inflammatory processes against antigens other than the parasite's, as previously suggested (26, 71). In contrast, a positive association between atopy and *S. mansoni* infection has been reported in a fishing community of Uganda highly exposed to multiple parasite infections and with high prevalence of allergen-reactive IgE (35), thus suggesting that the effect of helminth infection may be influenced by the parasite burden, time and chronicity of the infection, and co-infection with other species.

Although the modulatory mechanisms induced by helminth infection that are capable of regulating allergic reactivity are not fully understood, experimental data obtained from mice models infected with *S. mansoni* or immunized with schistosome antigens and submitted to ovalbumin-induced asthma (72–74) and studies with asthma patients living in poly-helminthic endemic areas (75) showed that modulation of asthma inflammation was accompanied by induction of Treg and/or IL-10 production and reduction of Th-2 cytokine production, mainly IL-4 and IL-5, eosinophilia, and IgE levels. In the current study, we measured the concentration of different immune mediators in serum or blood samples without further stimulation. Most of the individuals showed undetectable levels of cytokines in the serum, and the categorization of the individuals as non-responders, and high- or low-responders for each immune mediator allowed us to show that the frequency of IL-10-, TNF-α- and IL-33-responders was higher among IgE-reactive individuals. Additionally, we demonstrated that allergic reactivity was positively associated with high levels of IL-33 or TNF-α, but low levels of IL-10, thus indicating a less-modulated type-2 inflammatory response among the atopic population. The role of IL-10 production in the regulatory network induced by helminth infection, including *Schistosoma*, has been pointed out by many (24, 75). Moreover, the severity of allergic diseases and the susceptibility to helminth infection has been associated with some polymorphic forms of the IL-10 gene (66, 76).

Regarding IL-33, it is well known that this alarmin is produced mainly by the endothelium, epithelium, and fibroblasts, and when secreted after cell damage, it binds to its receptors (ST2) expressed in innate immune cells, such as ILC2, and stimulates the early production of IL-13 and IL-5, which leads to eosinophil infiltration and activation of Th2 response (4, 77, 78). Proteases from helminths and from allergens can promote epithelial damage and secretion of IL-33, which is responsible for potentiating Th-2 inflammation (4), thus indicating that IL-33/ST2 activation is related to type-2 inflammation intensity in epithelial barriers (79). Moreover, IL-33 levels correlate with clinical asthma severity (80), and IL-33 or its receptor (ST2) gene polymorphic variants have been implicated in susceptibility to allergic rhinitis (81) and the risk of asthma (82–84). Similarly, the positive association of allergic reactivity with the production of TNF-α demonstrated in the present study may also be related to the pro-inflammatory role of this cytokine in allergic processes, acting to amplify the type-2 inflammation (85). More recently, Choi et al. (86) demonstrated that house dust mite (HDM)-derived chitin induces TNF-α production, which is a key mediator in the development of Th2-cell response to inhaled allergens (86). Therefore, the positive association of IL-33 or TNF-α high-responders and atopy showed in the current study may reflect a non-modulated type-2 immune response.

The number of circulating eosinophils and the concentration of total IgE in serum was high in individuals living in Brejo do Amparo, independently of the allergic status. Similar results were also reported by Cooper et al. (34) in areas with high exposition to helminth infection (34). Despite the association observed in the univariate analysis, the total concentration of IgE was not associated with allergic reactivity in our multivariate model. However, it is important to consider that helminth-induced polyclonal IgE activation and/or the increase of parasite-induced IgG to carbohydrate determinants in glycoproteins that cross-react with environmental allergens may block allergic hypersensitivity reaction, thus helping with the modulation of allergic diseases symptoms in chronically exposed individuals (12, 87). These mechanisms may be taking place to modulate immune response in *S. mansoni*-infected individuals and should be better evaluated in the human population.

Finally, the extensive characterization of *S. mansoni* active infection by a combination of parasitological and molecular tests allowed us to identify a large number of individuals with very low parasite burden. The multivariate logistic regression model confirmed that, while *S. mansoni*-infected individuals with higher parasite burden (>12 EPG) showed risk of IgE-reactivity 6 times lower, individuals infected with very low parasite burden (≤12 EPG) presented allergic IgE-reactivity similar to non-infected individuals. This is the first time that a minimum threshold of parasite load has been identified as required for modulation of allergic response. The importance of helminth loading for induction of allergic modulation has been suggested by Oliveira et al. (51) in a rural area in northeastern Brazil; and, although they showed that individuals infected with *S. mansoni* presented reduced risks of developing allergic diseases even with low parasite load, their diagnostic was based on Kato-Katz only, and it is very unlikely that it included individuals with ≤12 EPG (52). Rujeni et al. (88), on the other hand, showed that *S. hematobium* infection was negatively associated with atopic response only in individuals living in a Zimbabwean village of high transmission, but not for individuals living in a low-transmission village with similar environment conditions, which correlates well with our own results (88).

Although we were able to show the effect of *S. mansoni* parasite burden and immune response on allergic reactivity, an important methodological limitation inherent of cross-sectional studies is the impossibility of establishing causal inferences. Further cohort studies are required to better elucidate the causal relationship of these effects.

In conclusion, herein we showed that *Schistosoma* burden is essential for the modulatory effect of allergic reactivity, even in an endemic area where the population is frequently exposed to infection. Besides the inverse correlation between parasite burden and IgE-reactivity, our data clearly show, for the first time, that very low parasite loads (≤12 EPG) are not enough to trigger modulatory mechanisms and, thus, do not affect the prevalence and intensity of the allergic responses. Even though we do not fully understand how *S. mansoni* infection modulate the allergic response, the current data indicated that the helminth infection, in a burden dependent fashion, induced anti-inflammatory response, including IL-10 production, that reduced IL-33 and/or TNF-α responses, which are associated with decreased IgE-reactivity. These results represent an important contribution to the understanding of helminthic-induced immunomodulatory mechanisms and should be further explored in the search for novel therapeutic strategies for the treatment of both helminthic infections and allergic diseases.

## Author Contributions

SR, SG, and DN-C: Conceptualization. SR, FM, JR-O, VC, CS, EO, and SG: Performed experiments. SR, FM, EO, MC, and DN-C: Data analysis. MC, SG, and DN-C: Supervision. SG and DN-C: Resources and project administration. SR and DN-C Writing the manuscript. All the authors reviewed and approved the manuscript.

### Conflict of Interest Statement

The authors declare that the research was conducted in the absence of any commercial or financial relationships that could be construed as a potential conflict of interest.
